# Analyzing intrinsic plasmonic chirality by tracking the interplay of electric and magnetic dipole modes

**DOI:** 10.1038/s41598-017-11571-9

**Published:** 2017-09-11

**Authors:** Li Hu, Yingzhou Huang, Lujun Pan, Yurui Fang

**Affiliations:** 1 Chongqing Engineering Laboratory for Detection, Control and Integrated System, School of Computer Science and Information Engineering, Chongqing Technology and Business University, Chongqing, 400067 P. R. China; 2 Soft Matter and Interdisciplinary Research Center, College of Physics, Chongqing University, Chongqing, 400044 P. R. China; 3 Department of Physics, Chalmers University of Technology, Gorthenburg, SE41296 Sweden; 4 Present Address: Key Laboratory of Materials Modification by Laser, Electron, and Ion Beams (Ministry of Education), School of Physics, Dalian University of Technology, Dalian, 116024 P. R. China

## Abstract

Plasmonic chirality represents significant potential for novel nanooptical devices due to its association with strong chiroptical responses. Previous reports on plasmonic chirality mechanism mainly focus on phase retardation and coupling. In this paper, we propose a model similar to the chiral molecules for explaining the intrinsic plasmonic chirality mechanism of varies 3D chiral structures quantitatively based on the interplay and mixing of electric and magnetic dipole modes (directly from electromagnetic field numerical simulations), which forms mixed electric and magnetic polarizability.

## Introduction

Objects that cannot be superimposed with their mirror image are chiral. Chirality is a common concept in nature^1, 2^. Circular dichroism (CD), the difference in absorption of left circularly polarized (LCP) light and right circularly polarized (RCP) light by chiral molecules, is very sensitive to molecular conformations and chirality. CD spectroscopy is a powerful analytical tool with a wide variety of uses ranging from the determination of sugar concentration in wine to the quality control of pharmaceutical processes. However, the molecular CD of natural compounds such as organic and biological chiral molecules is typically very weak and occurs in the ultraviolet region (150–300 nm), which substantially limits their applications. Proving the CD effect of chiral molecules beyond the UV region with large magnitudes has garnered a considerable amount of research interests. It has been recently suggested that plasmonic nanostructures can be utilized to boost the sensitivity of the CD measurement by generating super chiral electromagnetic near-fields or by exploiting their acute response to the immediate environment^3^. A lot of previous studies have demonstrated that artificial plasmonic nanostructures, such as chiral metal particle^4, 5^, DNA based self-assembled metal particles^6, 7^, helical nanowires^8–10^, etc^11–13^ can give rise to natural optical activity.

In the past several years, researchers have provided various explanations for the chiral mechanism such as the phase delay of two orthogonal directions of the structures^14^, the asymmetry of the structure to circularly polarized light (CPL)^15^, the resonant modes overlapping with the rotation direction of the CPL^16^, and the Born-Kuhn model^17^. All existing explanations for the CD effect of plasmonic structures are phenomenological, though a more general and profound explanation is analogic to the plasmonic structure as a chiral molecule^1^. The chiral mechanism in the chiral molecule model is obtained from an ab initio model in which the chiral effect originates from the interaction between electric and magnetic dipoles. Metal spirals (helices) as chiral meta-atoms for chiral materials have been studied with the concept many year ago^18, 19^. Plum borrowed this idea to qualitatively explain the extrinsic chirality of plasmonic nanostructures^20, 21^, as well as Govorov mentioned similar mechanism for the small chiral metal crystal^5^, and both are phenomenological description. Later, Tang borrowed Lipkin’ theory^22^ to describe the chirality of the chiral field quantitatively. Basically all of the plasmonic chirality can be understood with the quantitative polarizability and chiral field. In a study we previously conducted, we quantitatively analyzed the mechanism of *extrinsic* plasmonic chirality via the interplay of electric and magnetic dipole modes^23^. Despite many valuable contributions to the literature, there are currently a few direct quantitative models of the CD in *intrinsic* three-dimensional (3D) chiral plasmonic nanostructures^24–26^.

In this paper, using the coupled-dipole approximation (CDA) method in ref. 23, we first present a similar analytical model with non-orthogonal electric and magnetic dipoles to show the intrinsic chirality picture, which is different from the extrinsic chirality picture. The orientation of the electric and magnetic dipoles and the incident light vector make the chirality polarizability $G{\boldsymbol{\text{'}}}{\boldsymbol{\text{'}}}$ has a $-{\boldsymbol{i}}$ factor. Then using the model we analyze the plasmonic CD of intrinsic 3D chiral nanostructures directly with the results from the electromagnetic field simulations for Born-Kuhn model and a few different 3D plasmonic chiral nanostructures. The interplay of electric and magnetic modes of the nanostructure is analyzed and found to be responsible for the CD in dipole resonant range. The coupled electric and magnetic model and finite element method (FEM, COMSOL Multiphysics) analysis results agree with the CD quite well for the Born-Kuhn model; and also agree quite well for some other different 3D plasmonic chiral nanostructures in the dipole modes range. Higher order modes are out of the scope of our model, and there is some mismatch observed that is possibly caused by electric dipole-quadruple interaction. The proposed model and analytical method are expected to be extended to more 3D plasmonic chiral structures. The coupled electric-magnetic dipole interaction model and analysis method give a simple and easy way to track the mechanism and avoid complex detailed derivation process for plasmonic chiral structures.

## Results and Discussion

### Electric-magnetic dipoles interaction for *intrinsic* plasmonic chirality

It has been well established that the CD of a chiral molecule originates from the mixed interaction of electric-magnetic dipole moment and electric dipole-quadruple moment^1^. As plenty of molecules are randomly distributed, the electric dipole-quadruple interaction is typically cancelled during experimentation. Analogic to the chiral molecules, for the 3D chiral plasmonic structure, the CD effect is the result of the interaction of both electric (${\boldsymbol{e}}$)- magnetic (${\boldsymbol{m}}$) dipoles and electric dipole-quadruple (${\boldsymbol{q}}$) responses of the structure^1^. Higher order modes can usually be decomposed into three basic modes in different parts in simple chiral plasmonic structures. The electric quadruple mode is equivalent to the magnetic dipole mode in most of the chiral structure, so we mainly focused here on the interplay of the electric and magnetic dipole modes. The system consisted with electric dipole (${\boldsymbol{e}}$), magnetic dipole (${\boldsymbol{m}}$) and wave vector (${\boldsymbol{k}}$) constructs a 3D chiral configuration, which is asymmetric for LCP and RCP and results in CD effect. The interaction of the electric and magnetic dipoles will result in a mixed electric-magnetic polarizability ${\bf{G}}={\bf{G}}{\boldsymbol{^{\prime} }}+{\bf{i}}{\bf{G}}\text{'}\text{'}$, which makes the electric dipole moment ${{\bf{p}}}_{{\bf{e}}}$ and magnetic dipole moment ${{\bf{p}}}_{{\bf{m}}}$ as $\tilde{{{\boldsymbol{p}}}_{{\boldsymbol{e}}}}=\tilde{\alpha }\tilde{{\boldsymbol{E}}}-i\tilde{G}\tilde{{\boldsymbol{B}}},\,\tilde{{{\boldsymbol{p}}}_{m}}=\tilde{\chi }\tilde{{\boldsymbol{B}}}+i\tilde{G}\tilde{{\boldsymbol{E}}},\,\,$and CD is given by^2, 23^:

1 $${\rm{\Delta }}\sigma =G{\text{'}\text{'}}^{+}{C}^{+}-{G^{\prime\prime} }^{-}{C}^{-},$$

where $C=-\frac{{\varepsilon }_{0}\omega }{2}Im({{\boldsymbol{E}}}^{\ast }\cdot {\boldsymbol{B}})$ is the optical chirality, which can be calculated for any monochromatic electromagnetic field.

**Figure 1:**
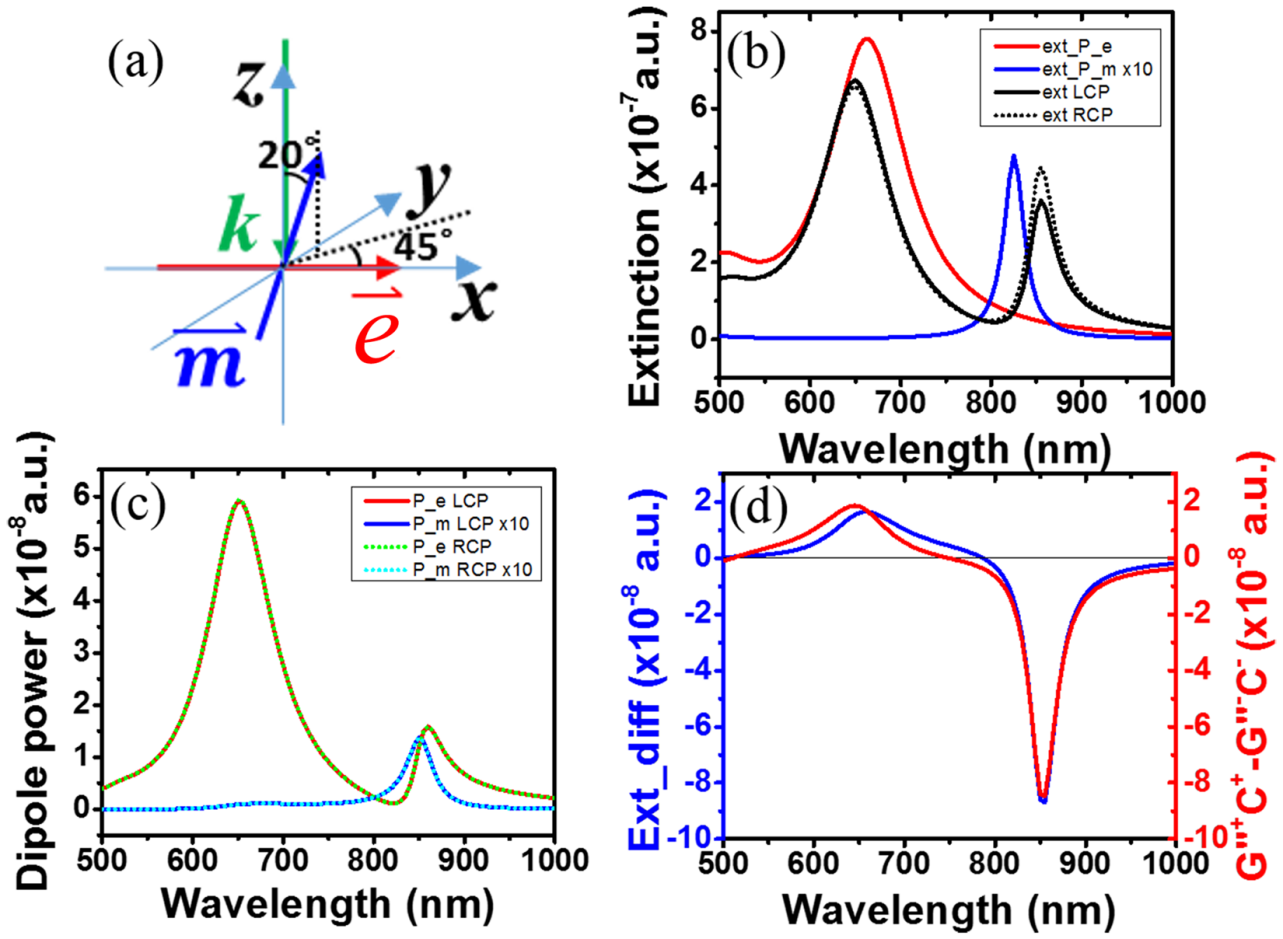
Coupled dipole approximation calculations for coupled electric dipole (ellipsoid a = 100 nm, b = c = 30 nm) and magnetic plasmonic dipole (ellipsoid a = 30 nm, b = c = 4.5 nm). (**a**) Schematics of the orientation of the wave vector, the coupled electric dipole and magnetic dipole. (**b**) Extinction spectra for uncoupled electric and magnetic plasmonic dipoles, and coupled electric and magnetic plasmonic dipoles under CPL illuminations. (**c**) Dipole power of the individual coupled electric and magnetic plasmonic dipoles. (**d**) Extinction difference (CD) of the coupled system (blue curve) and imaginary part of the mixed electric and magnetic polarizability of the coupled system.

${\boldsymbol{G}}$ is usually expressed as the dot product of ${\boldsymbol{e}}$ and ${\boldsymbol{m}}$ for a chiral molecule. It has been found that the interaction of electric and magnetic momentum gives ${\boldsymbol{G}}$ value for plasmonic chiral structures similar with chiral molecule. However, it is important to note here that ${\boldsymbol{G}}$ is usually unknown in plasmonic chiral structures. Principally, there are some ways to get the value for arbitrary objects^27, 28^. For some simple structures, ${\boldsymbol{G}}$ can be obtained from the scattered fields, but for most structures ${\boldsymbol{G}}$ is usually not easy to directly get (especially in complex structures in many previous studies, a chiral parameter κ was directly assumed to be a fixed value). Similar to the chiral molecule theory, it can be suggested that a direct dot product of electric and magnetic dipole modes (${\boldsymbol{e}}\cdot {\boldsymbol{m}}$) of the chiral plasmonic structures from numerical simulations can be used to express the ${\boldsymbol{G}}$ value. *For extrinsic chirality*, *the electric and magnetic dipoles have an orthogonal angle and the dipoles have zero projection onto each other*, *which causes a zero dot product for* ${\boldsymbol{e}}$ *and* ${\boldsymbol{m}}$. The interaction occurs with the assistance of a titled incident electromagnetic (EM) wave^21, 23^. *For intrinsic chirality*, *the electric and magnetic dipoles have a non-orthogonal angle and the dipoles have non-zero projection onto each other*, *which causes a non-zero dot product* (Fig. 1a). So it doesn’t need a titled incident wave to bring the interaction for ${\boldsymbol{e}}$ and ${\boldsymbol{m}}$. The illumination wave vector, induced electric mode and magnetic dipole mode *(* ***k***, ***e***, ***m*** *) are in the same plane for extrinsic chirality*, so the interaction of ***m*** and ***H*** has a π difference with the ***e*** and ***E*** interaction. Conversely, for intrinsic chirality, ${\boldsymbol{e}}$ and ${\boldsymbol{m}}$ can directly interact in the plane perpendicular to the wave vector ${\boldsymbol{k}}$, so they can interact with ***E*** and ***H*** simultaneously, and as a result *there is an* $i$ *factor difference between the plasmoic intrinsic and extrinsic chirality for* ${\boldsymbol{e}}$ *and* ${\boldsymbol{m}}$ *interaction*. So it is different from the extrinsic chirality we reported previously with $G^{\prime\prime}  \sim -Im({{\boldsymbol{p}}}_{{\boldsymbol{e}}}^{\ast }\cdot (-i{{\boldsymbol{p}}}_{{\boldsymbol{m}}}))$ ^23, 29, 30^, we identified the following intrinsic chirality polarizability here:

2 $$G^{\prime\prime}  \sim -Im({{\boldsymbol{p}}}_{{\boldsymbol{e}}}^{\ast }\cdot {{\boldsymbol{p}}}_{{\boldsymbol{m}}})$$

which is a result of the simultaneous excitation of electric and magnetic dipoles by in plane ***E*** and ***H*** components of the EM wave.

In the following part we analyzed the intrinsic plasmonic chiral molecule concept with the CDA model as shown in Fig. 1a with the formula we developed previously^23^. *The main difference from the previous molecule chiral molecule model is that* ${\boldsymbol{e}}$ *and* ${\boldsymbol{m}}$ *have non-orthogonal angle* (also see the methods part). Plasmonic structures allow the oscillating equivalent circular current in the structure to be treated as magnetons, and have as fast a response as the visible light frequency and equivalent negative magnetic susceptibility, which are known as chiral magnets^31^. Accordingly, with the appropriately arranged plasmonic structure, the induced dark mode acts like a strong resonant magneton and the magnetic susceptibility of the equivalent magnetic dipole $\overleftrightarrow{{\boldsymbol{u}}}$ can be treated like a plasmonic ellipsoid particle in the model. The uncoupled ***e*** and ***m*** dipoles momenta are shown in Fig. 1b; *they are pure* ***e*** *or* ***m*** *dipoles without the mixed polarizability* ***G***. When they are coupled together, the dipole momenta of the coupled system (Fig. 1c) clearly show *mixed polarizability;* both the resonant peaks have mixed electric and magnetic moments. This means there are interactions between electric and magnetic components in both electric and magnetic hybrid modes under incident EM wave.

The coupled system shows obvious CD effect, as expected, due to the mixed polarizability of the electric and magnetic dipoles (similar to that of chiral molecules). The extinction spectra under CPL is shown in Fig. 1b. The two peaks split and show a Fano profile with a dip right at the individual magnetic resonant wavelength due to the energy transfer between the ***e*** and ***m*** dipoles with phase delay, i.e., a typical Fano interference process. *As both CD and Fano can be caused by the interaction of* ***e*** *and* ***m*** *modes*, *there is possible Fano effect whenever there is CD effect*. The CD effect is directly characterized by the difference in extinction shown in Fig. 1d (blue curve). The CD calculated via molecule analysis Formulas 1 and 2 is shown in Fig. 1d (red curve). The two curves match each other very well, which indicates that the CD generation mechanism of the intrinsic plasmonic 3D chiral structures is indeed quantitatively determined and connected to the CD spectrum via the interaction of mixed of ***e*** and ***m*** dipole modes.

### Born-Kuhn model analyzing with numerical dipole model

**Figure 2:**
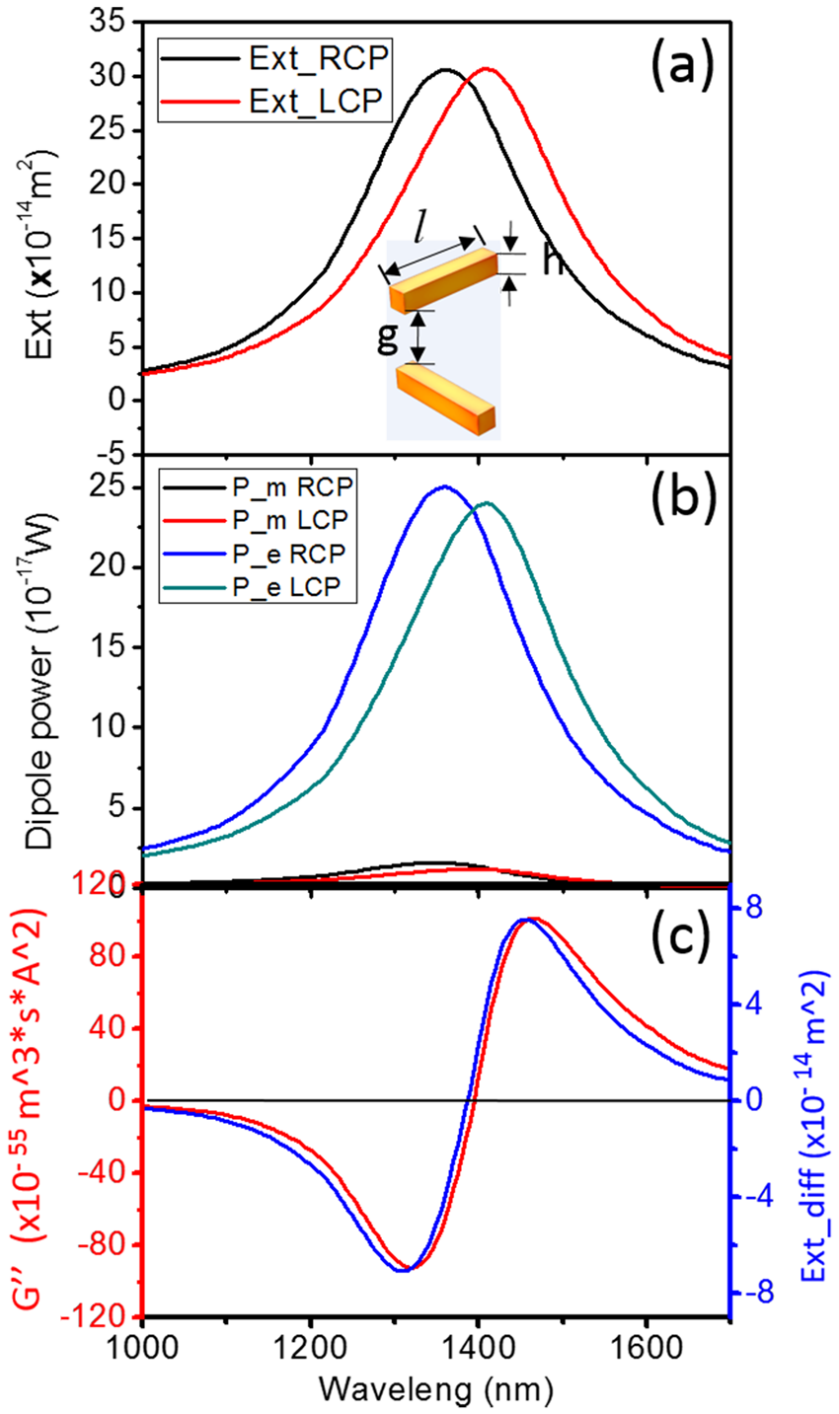
Coupled electric and magnetic dipole analysis for Born-Kuhn model. (**a**) Extinction spectra of the structure (inset) under LCP and RCP excited (length *l* = 223 nm, height *h* = 40 nm, width *w* = 40 nm, gap *g* = 120 nm). (**b**) Electric and magnetic dipoles power yielded by the structure under CPL illumination. (**c**) Imaginary part of the mixed electric and magnetic polarizability (red curve) and extinction difference (CD) of the coupled system (blue curve).

One of the most intuitive ways to understand the generation of natural optical activity in chiral media is the traditional coupled oscillator model of Born and Kuhn^17, 32, 33^ which consists of two identical, vertically displaced, coupled oscillators (Fig. 2a, inset). The plasmonic Born-Kuhn model was investigated here in the same manner as the mixed coupled dipole analytical model, but with electric and magnetic dipoles momenta obtained from numerical FEM results (Fig. 2). Figure 2a shows the extinction spectrum of the structure under LCP and RCP excitation. Figure 2b shows the electric and magnetic dipole moments. As discussed above, the electric and magnetic dipole resonances are mixed together, showing electric and magnetic components at the resonant peaks. The CD can then be obtained based on extinction difference; the mixed polarizability is obtained via Formula 1 and 2 (we only show $G^{\prime\prime}$ here, as the chiral fields ${C}_{CPL}$ for LCP and RCP have opposite signs, so $G^{\prime\prime}$ is the sum of ${{\boldsymbol{G}}}^{{\boldsymbol{\text{'}}}{\boldsymbol{\text{'}}}\pm }$). As shown in Fig. 2c, the CD spectrum calculated with Formula 2 (red curve) and the extinction difference (blue curve) are consistent. This validates our deduction of the quantitative analytical mechanism, and represents a step forward in better explaining the plasmonic 3D chiral structures.

### Numerical dipole interaction analyzing for different intrinsic chiral structures

**Figure 3:**
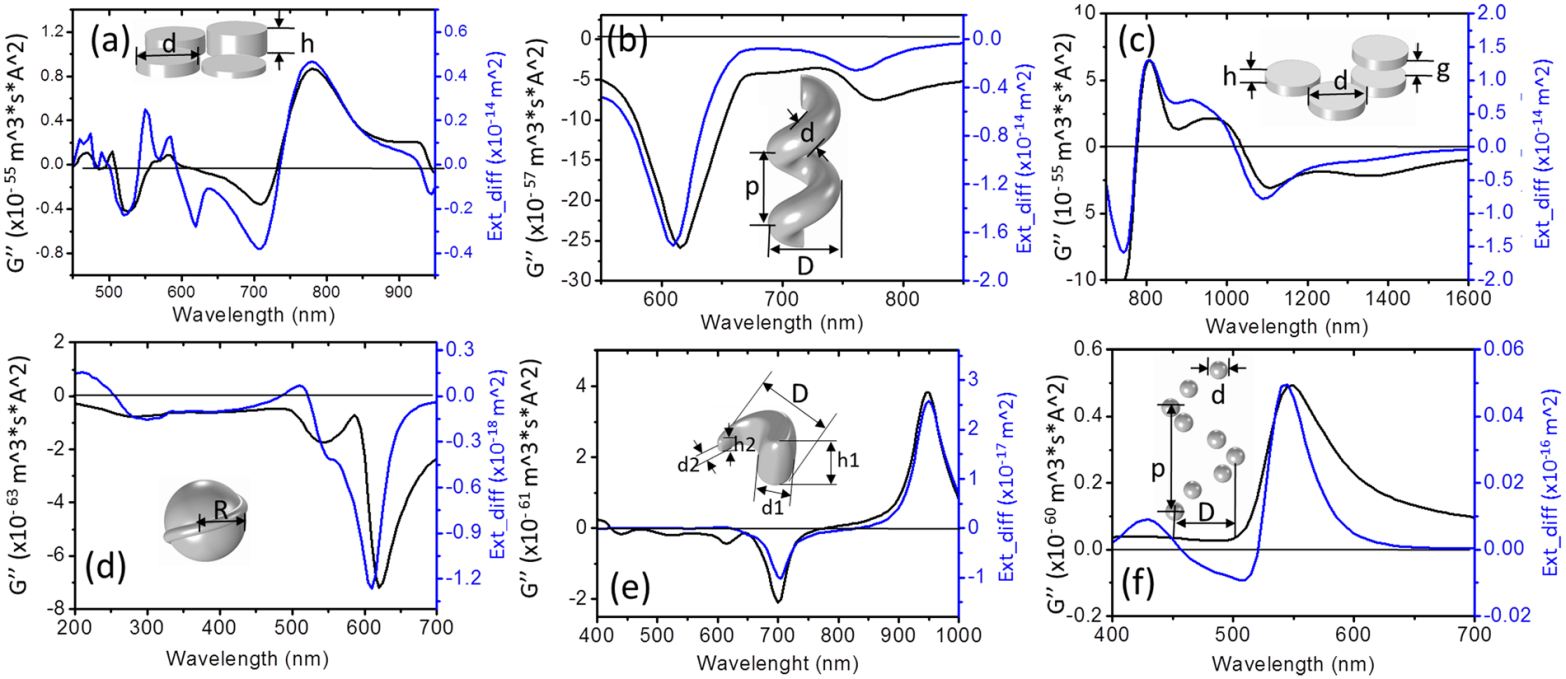
Comparison of CD spectra for coupled electric -magnetic dipole modes analysis and extinction difference of 3D plasmonic chiral structures. (**a**) Quasi-3D oligomers (d = 100 nm, h = 10 nm, 20 nm, 30 nm, 40 nm, gap between oligomers: 2 nm). (**b**) Plasmonic nanohelix (major diameter: D = 36 nm, minor diameter: d = 28 nm, helical pitch: p = 60 nm). (**c**) 3D chiral plasmonic oligomers (diameter: d = 100 nm, nanoparticle thickness: h = 40 nm, gap between the oligomers in bottom layer: s = 20 nm, gap between two layers: g = 70 nm). (**d**) Chiral nanocrystals (sphere radius: R = 7 nm, twister radius: 1 nm). (**e**) Spiral-type ramp nanostructures (outer diameter D = 22.5 nm, root diameter d1 = 11.3 nm, tip diameter d2 = 1.875 nm, root height h1 = 17.5 nm, tip height h2 = 2.5 nm). (**f**) Gold nanoparticle helices nanosphere (diameter: d = 10 nm, major diameter: D = 34 nm, helical pitch: p = 54 nm).

To further verify the above mechanism, six plasmonic chiral structures described in previous papers (see Supporting Information for details) were investigated with the proposed analytical model. The electric and magnetic dipole moments were obtained from FEM numerical results (see Methods part). The CD spectra from Formula 2 (black curve) and from the extinction difference (blue curve) are compared in Fig. 3. The quasi-three-dimensional chiral hetero tetramers was investigated first via the proposed analytical model (Fig. 3a), where the CD effect is due to strong near field coupling and intricate phase retardation effects^34^. As shown in Fig. 3a, the dipole modes match well but multiple modes do not, because for multiple modes there is more than one equivalent magnetic dipole oscillating out of phase but with delayed interaction with the electric modes, thus the whole effect is characterized by interference superposition. Figure 3b shows the CD of the plamonic nanohelix which was previously analyzed by discrete dipole approximation method and explained as the extinction combination of under x and y polarization with complementary phases^14, 35^. The CD spectra from Formula 2 and extinction difference anastomosed each other. Figure 3c shows the 3D arrangements of plasmonic “meta-atoms” which is formed due to the chiral configuration having caused helical displacement currents giving rise to an induced magnetic moment component parallel to the usual electric dipole moment and resulting a unique interaction of the meta-molecule with CPL^36^. The two curve match well. Figure 3d shows a comparison for a spherical nanoparticle with slight deformations. The CD mechanism has been explained as the mixing between plasmon harmonics with different angular momenta^5^; here the two curves match the main trend but with larger deviation possibly because other significant non-dipole modes were present. Figure 3e shows a 3D chiral nanocrescent where the CD originating from the rotation incident E field vector matches the arranged equivalent dipoles at the resonant modes^16^. The CD curve from the coupled dipole modes analysis matches the extinction result very well. For the self-assembled gold nanohelix shown in Fig. 3f, CD occurred due to the helical arrangement of the gold nanoparticles resulting in coupled plasmon waves propagating along a helical path and causing increased absorption of those components of the incident light that are in accordance with the handedness of the helices^37^. The spectrum of G” and extinction difference were largely the same, despite slight mismatch that may have originated from the quadruple modes being arranged by the small particle dipole (which cannot be entirely treated as a magnetic mode).

Taken together, these results show that the CD of 3D plasmonic structures can indeed be quantitatively analyzed according to the coupled e and m dipole analytical model. There are slight differences among a few dipole models due to the interaction of dipole and quadruple modes, where the quadruple mode cannot be entirely represented by a magnetic dipole.

### Scattering circular dichroism consideration

Now scattering measurements of the chiral structures take a large part of plasmonic chirality research, therefore it is useful to compare the scattering difference with the model. Because the radiation from the magnetic dipole is very weak, so it is can be ignored and the scattering can be expressed as

3 $${\rm{SCAT}}=\,\frac{{\omega }^{4}}{3{c}^{3}}{{\boldsymbol{p}}}_{{\boldsymbol{e}}}\cdot {{\boldsymbol{p}}}_{{\boldsymbol{e}}}^{\ast }\propto (\alpha {\boldsymbol{E}}-i\tilde{G}{\boldsymbol{B}})\cdot {(\alpha {\boldsymbol{E}}-i\tilde{G}{\boldsymbol{B}})}^{\ast }.$$

If we take away the ${{\boldsymbol{B}}}^{{\bf{2}}}$ item because it is very small, then

4 $${\rm{\Delta }}\text{SCAT}\propto \,i\,(\alpha {{\boldsymbol{E}}}^{+}{\tilde{G}}^{+}{{\boldsymbol{B}}}^{+}-{\tilde{G}}^{+}{{\boldsymbol{B}}}^{+}\alpha {{\boldsymbol{E}}}^{+})-i\,(\alpha {{\boldsymbol{E}}}^{-}{\tilde{G}}^{-}{{\boldsymbol{B}}}^{-}-{\tilde{G}}^{-}{{\boldsymbol{B}}}^{-}\alpha {{\boldsymbol{E}}}^{-}).$$

**Figure 4:**
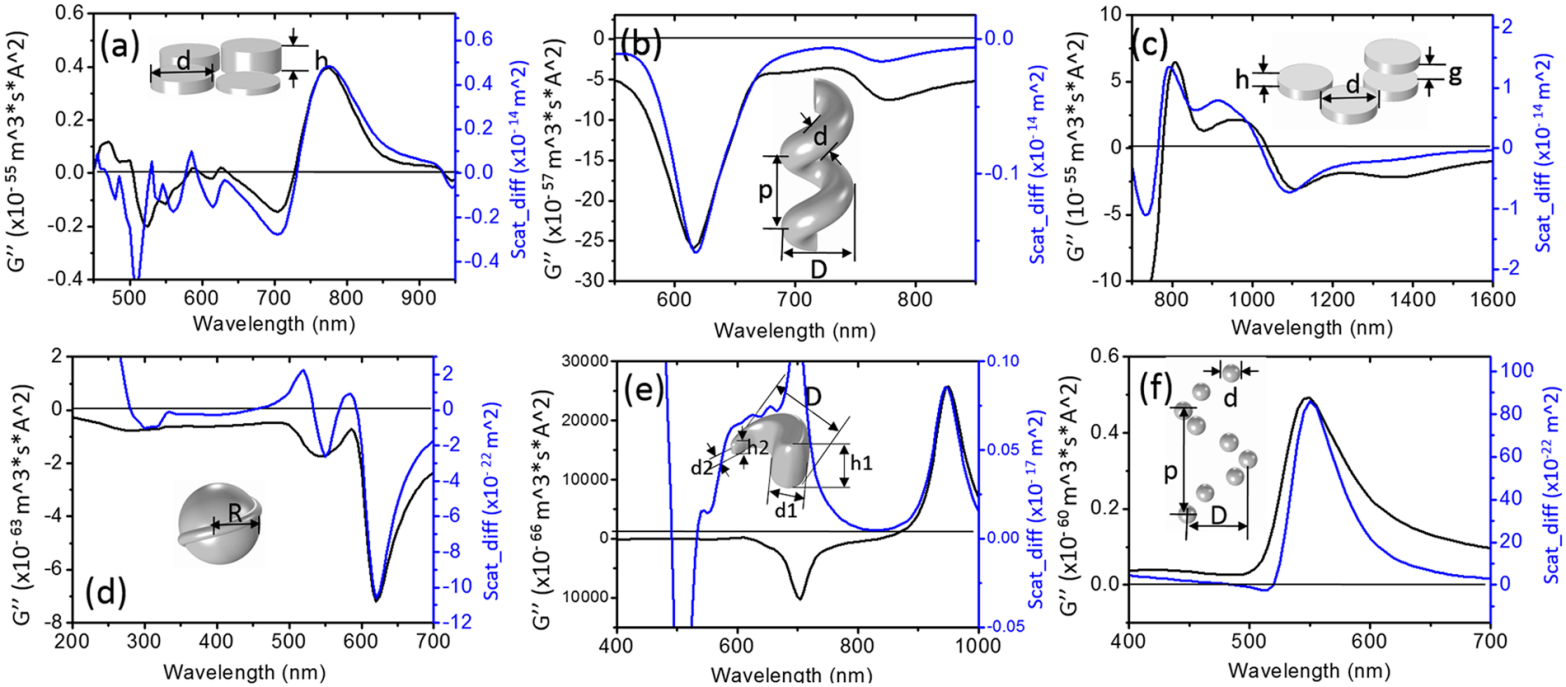
Comparison of CD spectra for coupled electric -magnetic dipole modes analysis and scattering difference of 3D plasmonic chiral structures that are the same with Fig. 3. (**a**) Quasi-3D oligomers. (**b**) Plasmonic nanohelix. (**c**) 3D chiral plasmonic oligomers. (**d**) Chiral nanocrystals. (**e**) Spiral-type ramp nanostructures. (**f**) Gold nanoparticle helices nanosphere.

Here the scattering circular dichroism is directly related to $\tilde{G}$. According to equ. (2), $\tilde{G} \sim {{\boldsymbol{p}}}_{{\boldsymbol{e}}}^{\ast }\cdot {{\boldsymbol{p}}}_{{\boldsymbol{m}}}$. Usually $G^{\prime}$ is related to the optical rotatory dispersion (ORD), thus somehow is still related to the scattering. From this sense, $G^{\prime\prime}$ cannot directly reflect the scattering CD, however, we can still get the scattering CD profile from the model. In Fig. 4 the scattering CD is plotted together with $G^{\prime\prime}$, and one can find quite good agreement with the scattering CD, which is quite similar with that in Fig. 3 but with a smaller scattering difference value (see Supporting Information for details). Figure 4e has a large deviation at the 700 nm peak, which needs further investigation in the future. Generally speaking, for plasmonic structures, when the size is very small, the absorption is stronger than scattering; when the size is large, the scattering will take the main part of the extinction. So for the large chiral structures, the model can be used to analysis the scattering CD, for the small chiral structures, the model somehow still reflects part of the scattering CD properties. More detailed relation for the scattering CD and the model needs further investigation.

## Conclusion

In summary, the strong interplay between the non-orthogonal electric and magnetic dipole modes appears to result in the CD of plasmonic *intrinsic* chiral structures. The modes represented by ***e*** and ***m*** dipoles can be in certain sense used to quantitatively analyze the CD mechanism with the imaginary part of mixed ***e-m*** polarizability ***G***. The mixed polarizability originates from the strong interplay of electric and magnetic dipoles, which can be expressed with the dot product of the two dipoles. In this way, one can avoid the complex derivation process for chiral structures and easily focus on the interaction mechanism. The model is presented with CDA and verified with numerical FEM results in the Born-Kuhn model (a typical chiral model) and other 3D plasmonic chiral nanostructures. It is not suitable for higher order modes. Mismatch appears for some structures due to the unignorable interaction of dipole and quadrupole modes, which merits further research. The proposed model is expected to be extended to more 3D plasmonic chiral structures. The techniques for obtaining mixed polarizability G for intrinsic and extrinsic plasmonic chirality^23^ are characterized by a $-i$ factor, likely because the magnetic mode is excited by the H field with different phases; this phenomenon also merits further research. The $G{}^{^{\prime} }{}^{^{\prime} }$ also reflects the scattering CD to some extent, not as obvious as the CD spectra. The coupled electric-magnetic dipole interaction model and analysis method give a simple and easy way to track the mechanism and avoid complex detailed derivation process for plasmonic chiral structures.

## Methods

### FEM Simulation

All full wave numerical simulations were done by using finite element method (FEM, commercial software package, Comsol Multiphysics 5.0). The three dimensional (3D) chiral nanostructrue were put in a homogeneous surrounding medium or on substrate. Non-uniform meshes were used for formatting the object. The largest mesh was set less than $\lambda /6$. Perfect matched layer (PML) was used to minimize the scattering from the outer boundary. The incident light was set to 1 V/m and propagates along the z axis. The total scattering cross sections were obtained by integrating the scattered power flux over an enclosed surface outside the 3D nanostructure, while the absorption cross sections were determined by integrating the Ohmic heating within the nanostructure. The circular dichroism of the system were calculated as the difference in extinction under left and right handed circularly polarized light ($CD={\sigma }_{L}-{\sigma }_{R}$). The electric and magnetic dipole moments were obtained from ${{\boldsymbol{p}}}_{{\boldsymbol{e}}}=\int {d}^{3}{r}^{{\boldsymbol{^{\prime} }}}{\bf{r}}\,{}^{{\boldsymbol{^{\prime} }}}\rho ({\bf{r}}\,{}^{{\boldsymbol{^{\prime} }}})$ and ${{\boldsymbol{p}}}_{{\boldsymbol{m}}}=\frac{1}{2}\int {d}^{3}{r}^{{\boldsymbol{^{\prime} }}}\,({\bf{r}}\,{}^{{\boldsymbol{^{\prime} }}}\times {\boldsymbol{J}})$, where ${\boldsymbol{\rho }}({\boldsymbol{r}}^{\prime} )$ is the charge density and ${\boldsymbol{J}}({\bf{r}}\,{}^{{\boldsymbol{^{\prime} }}})\,\,$ is the current density. The dipole power were from S16 & S17 in ref. 23. Because ${\boldsymbol{G}}$ is the mixed electric and magnetic polarizability in ${m}^{3}/\Omega$ units (corresponding to ${{\boldsymbol{\alpha }}}_{{\boldsymbol{em}}}[{m}^{3}]/{Z}_{0}[\Omega ]$). Consider the expression of $C=-{\varepsilon }_{0}\omega /2\ast Im({{\boldsymbol{E}}}^{\ast }\cdot {\boldsymbol{B}})$, $G\to G/{\varepsilon }_{0}$, in $\,{{\boldsymbol{\alpha }}}_{{\boldsymbol{em}}}[{m}^{3}]\ast c[m/s]$ units. For the purposes of our study we only used ${{\boldsymbol{p}}}_{{\boldsymbol{e}}}^{\ast }\cdot {{\boldsymbol{p}}}_{{\boldsymbol{m}}}$ which is in ${m}^{3}s{A}^{2}$ units. The chiral response for the molecule is basically $\kappa =4\pi {n}_{0}/3\ast (2/{\hbar}\sum [\omega /({\omega }_{jn}^{2}-{\omega }^{2})\ast (\langle n|{\boldsymbol{e}}|j\rangle \langle j|{\boldsymbol{m}}|n\rangle )])\,/{\varepsilon }_{0}$ (where ${n}_{0}$ is the volume density and $(2/{\hbar}\sum [\omega /({\omega }_{jn}^{2}-{\omega }^{2})]\,$is the density of states (DOS)) which indicates that $G=({{\boldsymbol{p}}}_{e}^{\ast }\cdot {{\boldsymbol{p}}}_{{\boldsymbol{m}}})\ast DOS=2/({\hbar}{Z}_{0}{c}^{2})\omega {{\boldsymbol{p}}}_{{\boldsymbol{e}}}^{\ast }\cdot {{\boldsymbol{p}}}_{{\boldsymbol{m}}}\ast [{{\boldsymbol{n}}}_{{\boldsymbol{u}}}\cdot {\boldsymbol{Im}}(\overleftrightarrow{{{\boldsymbol{G}}}_{{\boldsymbol{e}}}({{\boldsymbol{r}}}_{0},{{\boldsymbol{r}}}_{0};{\omega }_{0})}\cdot {{\boldsymbol{n}}}_{{\boldsymbol{m}}})]$, where $\overleftrightarrow{{{\boldsymbol{G}}}_{{\boldsymbol{e}}}}\,$has the relationship with electric field normal modes $\overleftrightarrow{{{\boldsymbol{G}}}_{{\boldsymbol{e}}}({\boldsymbol{r}},{\boldsymbol{r}}\,{\boldsymbol{^{\prime} }};\omega )}=\sum _{{\boldsymbol{k}}}{c}^{2}{{\boldsymbol{e}}}_{{\boldsymbol{k}}}^{\ast }({\boldsymbol{r}}{\boldsymbol{^{\prime} }},{\omega }_{{\boldsymbol{k}}}){{\boldsymbol{e}}}_{{\boldsymbol{k}}}({\boldsymbol{r}},{\omega }_{{\boldsymbol{k}}})/({\omega }_{{\boldsymbol{k}}}^{2}-{\omega }^{2})$ ^38^. To simplify the FEM simulations, we only calculated ${{\boldsymbol{p}}}_{{\boldsymbol{e}}}^{\ast }\cdot {{\boldsymbol{p}}}_{{\boldsymbol{m}}}$, which did not affect our understanding of the mechanism.

### Coupled-Dipole Approximation (CDA)

The coupled electric and magnetic dipoles with a non-orthogonal angle and having non-zero projection onto each other are calculated following ref. 23. The polarizability of the electric dipole $\overleftrightarrow{{{\boldsymbol{\alpha }}}_{1}}$ is set as a gold ellipsoid particle (a = 100 nm, b = c = 30 nm) with the long axis along **x**. The polarizability of the magnetic dipole $\overleftrightarrow{{{\bf{u}}}_{2}}$ is set on the basis of an ellipsoid particle (a = 30 nm, b = c = 4.5 nm) with the long axis along an azimuth angle by 20° to **z** axis and 45° to **x** axis (Fig. 1a). Because nature material has bad magnetic response in optical frequency and the magnetons in our paper are yield by the plasmon resonance with circular current, we used a fake ${{\boldsymbol{u}}}_{{\boldsymbol{particle}}}\,$value to yield the magnetic resonance in optical wavelength range, which is from ${{\boldsymbol{\varepsilon }}}_{{\boldsymbol{Au}}}$ but with imaginary part divided by 1.5 (the magnetic mode is dark, so the spectrum profile is narrower). The extinction under CPL can be obtained from ${\sigma }^{\pm }=\frac{\omega }{2}Im({{\boldsymbol{E}}}^{\ast }\cdot {{\boldsymbol{p}}}_{{\boldsymbol{e}}}+{{\boldsymbol{B}}}^{\ast }\cdot {{\boldsymbol{p}}}_{{\boldsymbol{m}}})$. The dipole power were from S16 & S17 in ref. 23.

## Electronic supplementary material

Supplementary information
