# Supplementary material for: Analyzing intrinsic plasmonic chirality by tracking the interplay of electric and magnetic dipole modes
Source: Sci Rep. 2017 Sep 11;7:11151. doi: 10.1038/s41598-017-11571-9 (PMC5593961; doi:10.1038/s41598-017-11571-9)
Supplement: Supplementary file 1 — Supplementary information [file 41598_2017_11571_MOESM1_ESM.pdf]

Supplementary information for

Analyzing intrinsic plasmonic chirality by tracking the interplay of electric and magnetic dipole modes

Li Hu[1,2], Yingzhou Huang[2], Lujun Pan[3] and Yurui Fang[3,4*]

[1] Chongqing Engineering Laboratory for Detection, Control and Integrated System, School of Computer Science and Information Engineering, Chongqing Technology and Business University, Chongqing, 400067, P. R. China

[2]Soft Matter and Interdisciplinary Research Center, College of Physics, Chongqing University, Chongqing, 400044, P. R. China

[3]Key Laboratory of Materials Modification by Laser, Electron, and Ion Beams (Ministry of Education), School of Physics and Optoelectronic Engineering, Dalian University of Technology, Dalian 116024, P. R. China

[4]Department of Physics, Chalmers University of Technology, Gorthenburg SE41296, Sweden

*Corresponding Author's Email: yrfang@dlut.edu.cn or fang.yurui@gmail.com (Y. Fang)

The mixed electric and magnetic dipole polarizability

Comparing S13 & S14 in ref. [1]

$$\boldsymbol{p_e} = \frac{\varepsilon_0 \overleftrightarrow{\boldsymbol{\alpha_1}} \boldsymbol{E_{1,in}} - Z_0 k^2 \varepsilon_0 \overleftrightarrow{\boldsymbol{\alpha_1}} \overleftrightarrow{\boldsymbol{G_m}}(\boldsymbol{r_m}, \boldsymbol{r_e}) \overleftrightarrow{\boldsymbol{u_2}} \boldsymbol{H_{2,in}}}{\overleftrightarrow{\boldsymbol{I}} + \boldsymbol{c} Z_0 k^4 \varepsilon_0 \overleftrightarrow{\boldsymbol{\alpha_1}} \overleftrightarrow{\boldsymbol{G_m}}(\boldsymbol{r_m}, \boldsymbol{r_e}) \overleftrightarrow{\boldsymbol{u_2}} \overleftrightarrow{\boldsymbol{G_m}}(\boldsymbol{r_e}, \boldsymbol{r_m})},$$

$$\boldsymbol{p_m} = \frac{\overleftrightarrow{\boldsymbol{u_2}} \boldsymbol{H_{2,in}} + \boldsymbol{c} k^2 \varepsilon_0 \overleftrightarrow{\boldsymbol{u_2}} \overleftrightarrow{\boldsymbol{G_m}}(\boldsymbol{r_e}, \boldsymbol{r_m}) \overleftrightarrow{\boldsymbol{\alpha_1}} \boldsymbol{E_{1,in}}}{\overleftrightarrow{\boldsymbol{I}} + \boldsymbol{c} Z_0 k^4 \varepsilon_0 \overleftrightarrow{\boldsymbol{u_2}} \overleftrightarrow{\boldsymbol{G_m}}(\boldsymbol{r_e}, \boldsymbol{r_m}) \overleftrightarrow{\boldsymbol{\alpha_1}} \overleftrightarrow{\boldsymbol{G_m}}(\boldsymbol{r_m}, \boldsymbol{r_e})}$$

with $\widetilde{\boldsymbol{p_e}} = \tilde{\alpha} \widetilde{\boldsymbol{E}} - i \tilde{G} \widetilde{\boldsymbol{B}}$, $\widetilde{\boldsymbol{p}_m} = \tilde{\chi} \widetilde{\boldsymbol{B}} + i \tilde{G} \widetilde{\boldsymbol{E}}$, we can get

$$\tilde{\alpha} = \frac{\varepsilon_0 \overleftrightarrow{\boldsymbol{\alpha_1}}}{\overleftrightarrow{\boldsymbol{I}} + \varepsilon_0 \boldsymbol{c} Z_0 k^4 \overleftrightarrow{\boldsymbol{\alpha_1}} \overleftrightarrow{\boldsymbol{G_m}}(\boldsymbol{r_m}, \boldsymbol{r_e}) \overleftrightarrow{\boldsymbol{u_2}} \overleftrightarrow{\boldsymbol{G_m}}(\boldsymbol{r_e}, \boldsymbol{r_m})} \tag{S1}$$

$$\tilde{\chi} = \frac{\overleftrightarrow{\boldsymbol{u_2}} / \boldsymbol{u_0}}{\overleftrightarrow{\boldsymbol{I}} + \varepsilon_0 \boldsymbol{c} Z_0 k^4 \overleftrightarrow{\boldsymbol{u_2}} \overleftrightarrow{\boldsymbol{G_m}}(\boldsymbol{r_e}, \boldsymbol{r_m}) \overleftrightarrow{\boldsymbol{\alpha_1}} \overleftrightarrow{\boldsymbol{G_m}}(\boldsymbol{r_m}, \boldsymbol{r_e})} \tag{S2}$$

$$\tilde{G} = -i \frac{\frac{\varepsilon_0 Z_0 k^2 \overleftrightarrow{\boldsymbol{\alpha_1}} \overleftrightarrow{\boldsymbol{G_m}}(\boldsymbol{r_m}, \boldsymbol{r_e}) \overleftrightarrow{\boldsymbol{u_2}}}{\boldsymbol{u_0}}}{\overleftrightarrow{\boldsymbol{I}} + \varepsilon_0 \boldsymbol{c} Z_0 k^4 \overleftrightarrow{\boldsymbol{\alpha_1}} \overleftrightarrow{\boldsymbol{G_m}}(\boldsymbol{r_m}, \boldsymbol{r_e}) \overleftrightarrow{\boldsymbol{u_2}} \overleftrightarrow{\boldsymbol{G_m}}(\boldsymbol{r_e}, \boldsymbol{r_m})} = \left(-i \frac{\varepsilon_0 \boldsymbol{c} k^2 \overleftrightarrow{\boldsymbol{u_2}} \overleftrightarrow{\boldsymbol{G_m}}(\boldsymbol{r_e}, \boldsymbol{r_m}) \overleftrightarrow{\boldsymbol{\alpha_1}}}{\overleftrightarrow{\boldsymbol{I}} + \varepsilon_0 \boldsymbol{c} Z_0 k^4 \overleftrightarrow{\boldsymbol{u_2}} \overleftrightarrow{\boldsymbol{G_m}}(\boldsymbol{r_e}, \boldsymbol{r_m}) \overleftrightarrow{\boldsymbol{\alpha_1}} \overleftrightarrow{\boldsymbol{G_m}}(\boldsymbol{r_m}, \boldsymbol{r_e})} \right)^* \tag{S3}$$

Consider the expression of $C = -\frac{\varepsilon_0 \omega}{2} Im(\boldsymbol{E}^* \cdot \boldsymbol{B})$, $G \to \frac{G}{\varepsilon_0}$.

The spectra of 3D chiral models

The extinction spectra, the dipole power spectra and CD spectra of the 3D chiral models and corresponding electric dipole momentum **Pe** and magnetic dipole momentum **Pm** in real part were shown in Fig S1-S13. The nanostructure were shown in the inset of the extinction spectra, respectively. All the structure were 3D chiral plasmonic structure because we expected to investigate the applicability of the quantitative model. The results of the simulation have a few difference with the published paper may be because that the structure were not in full accord or the meshes in simulation had some difference.

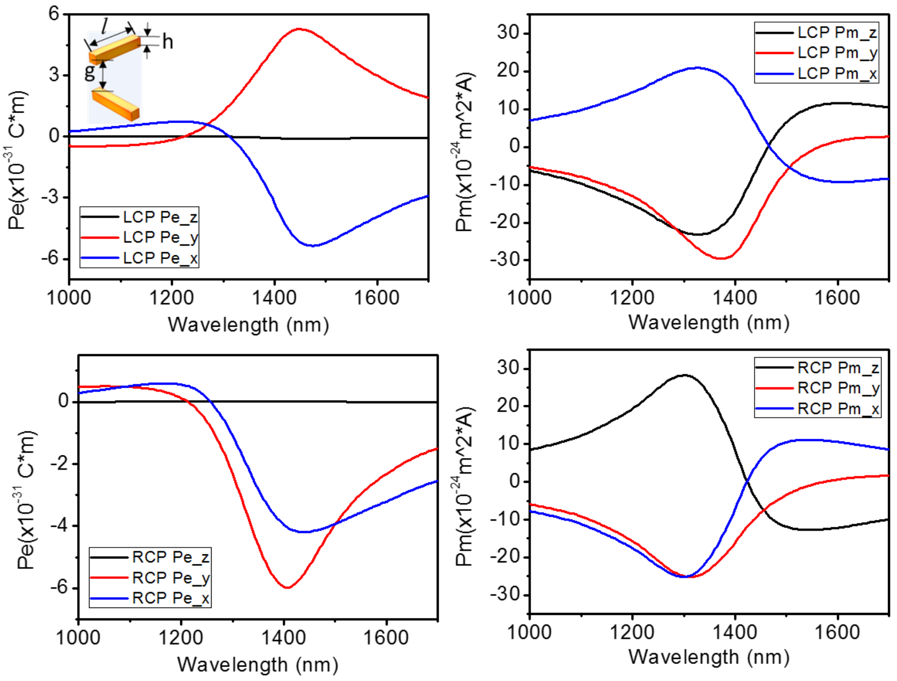

Figure S1. The electric dipole momentum $\mathbf{P_e}$ and magnetic dipole momentum $\mathbf{P_m}$ plotted in their x, y, z components with only real part for the structure in Fig. 2. The

figure is to show that the $\mathbf{P_e}$ and $\mathbf{P_m}$ have non zero components when they projects to each other, so the dot product is non zero.

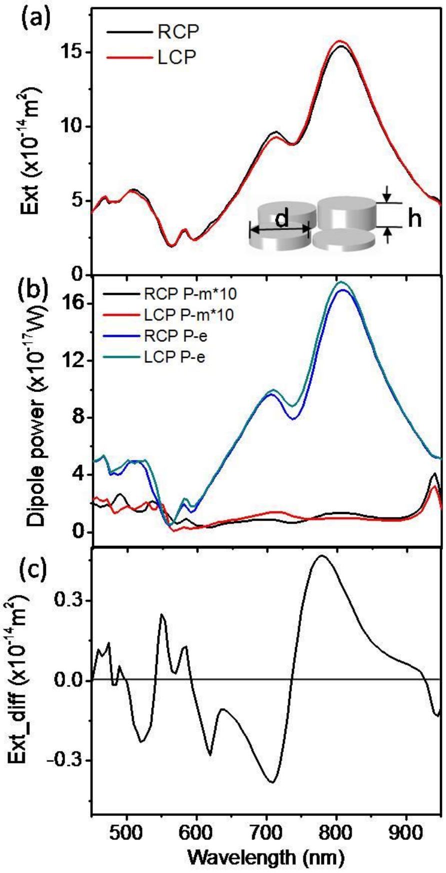

Figure S2.The extinction spectra (a), the dipole power spectra (b)and the extinction different (CD) spectra (c) of the Ag quasi-three-dimensional oligomers.(diameter: d=100nm, h=10 nm, 20 nm, 30 nm, 40 nm, the gap between the oligomers:2 nm)[2].

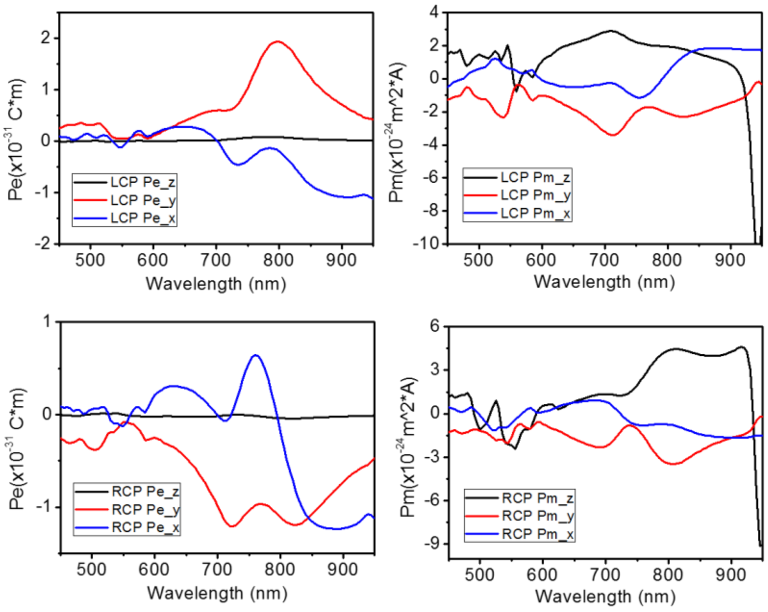

Figure S3. The electric dipole momentum $\mathbf{P_e}$ and magnetic dipole momentum $\mathbf{P_m}$ plotted in their x, y, z components with only real part for the structure in S2.

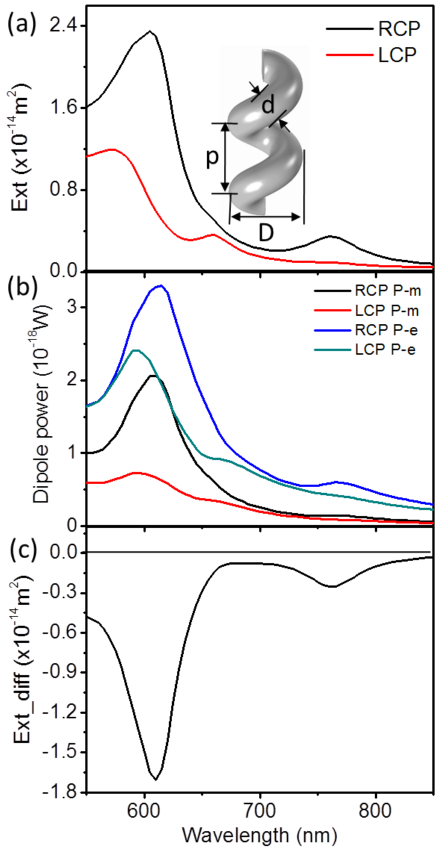

Figure S4 The extinction spectra (a), the dipole power spectra (b) and the extinction

different (CD) spectra (c) of the Cu plasmonicnanohelix. (major diameter: D=36 nm, minor diameter: d=28 nm, helical pitch: p= 60 nm)[3].

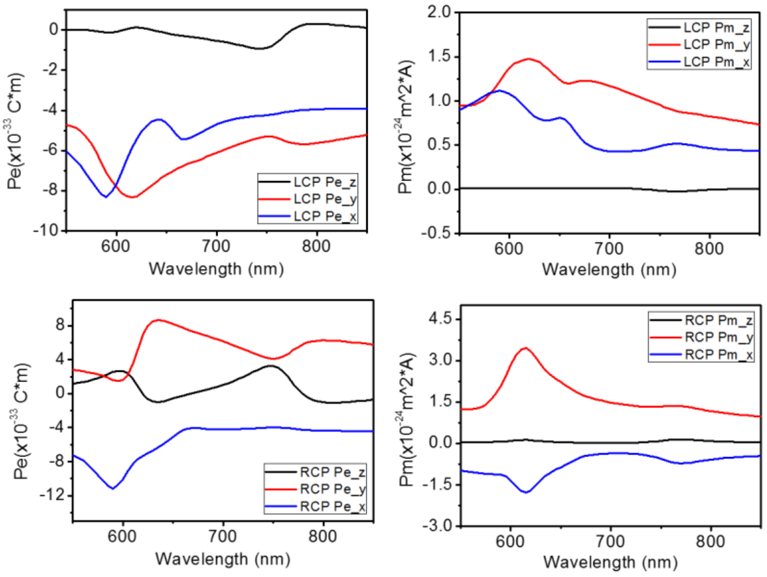

Figure S5. The electric dipole momentum $\mathbf{P_e}$ and magnetic dipole momentum $\mathbf{P_m}$ plotted in their x, y, z components with only real part for the structure in S4.

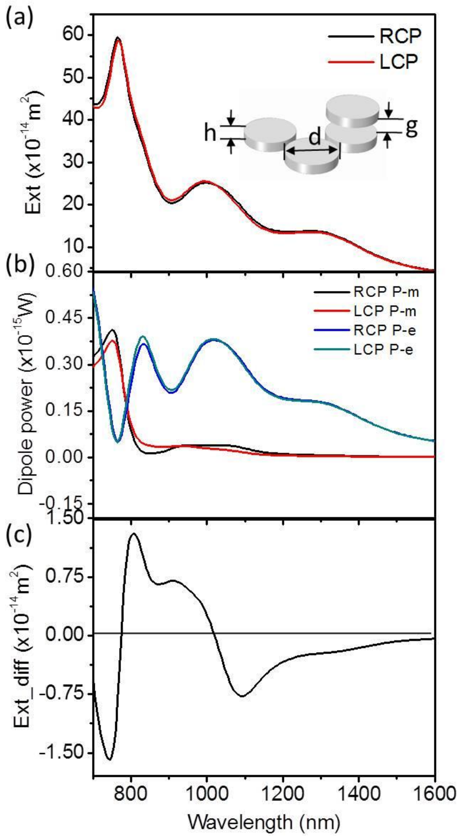

Figure S6. The extinction spectra (a), the dipole power spectra (b) and the extinction different (CD) spectra (c) of the Au 3D chiral plasmonic oligomers. (diameter:d=100 nm, thickness of nanoparticle: h=40nm, the gap between theoligomers in borrom layer: s=20 nm, the gap of two layers: g=70nm)[4].

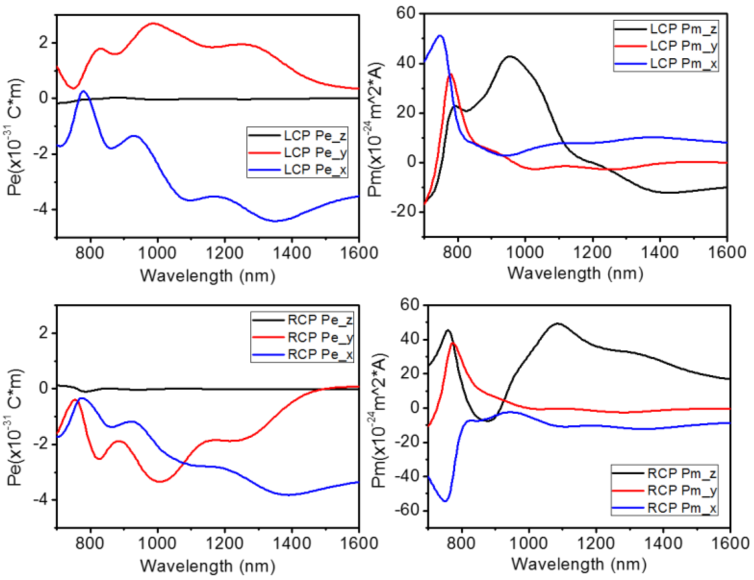

Figure S7. The electric dipole momentum $\mathbf{P_e}$ and magnetic dipole momentum $\mathbf{P_m}$ plotted in their x, y, z components with only real part for the structure in S6.

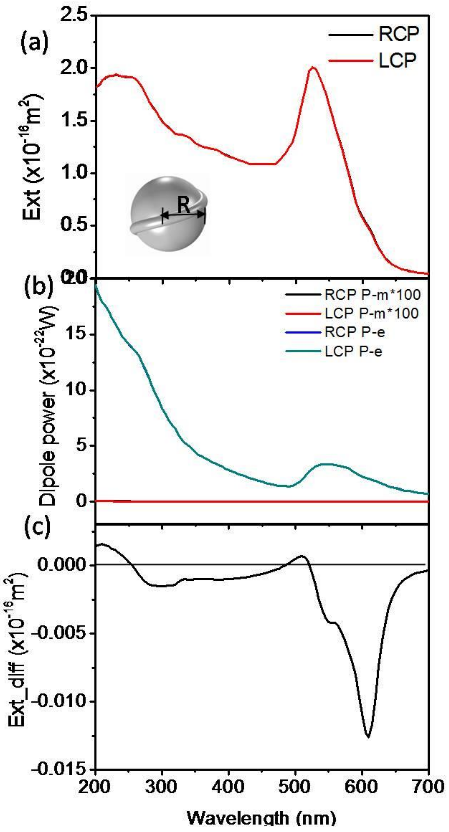

Figure S8. The extinction spectra (a), the dipole power spectra (b) and the extinction different (CD) spectra (c) of the Au chiral nanocrystals.(radius of sphere: R=7 nm, radius of the twiser: 1 nm)[5]

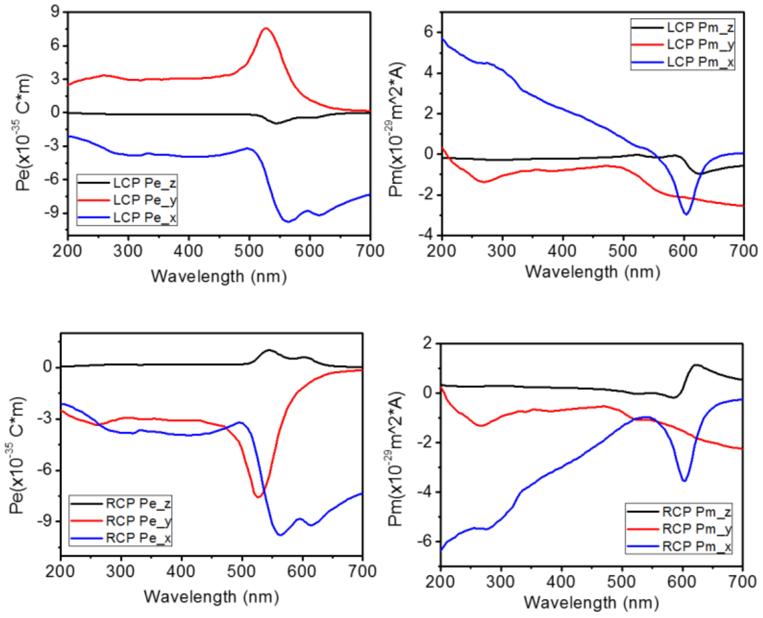

Figure S9. The electric dipole momentum $\mathbf{P_e}$ and magnetic dipole momentum $\mathbf{P_m}$ plotted in their x, y, z components with only real part for the structure in S8.

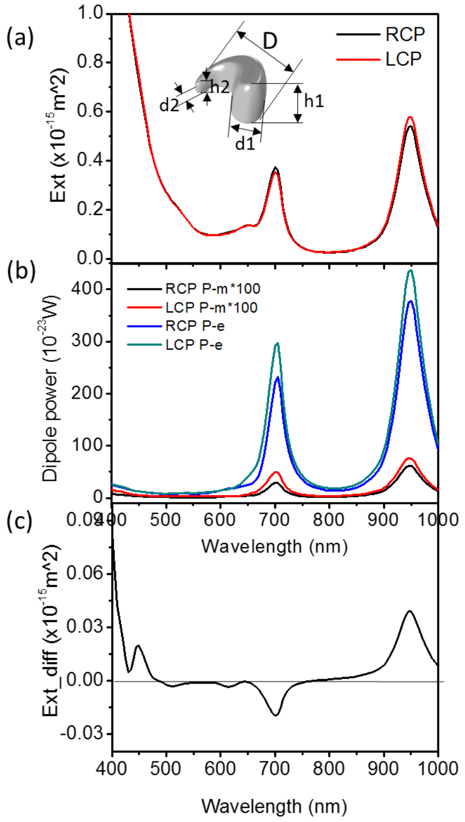

Figure S10. The extinction spectra (a), the dipole power spectra (b) and the extinction

different (CD) spectra (c) of the spiral-type ramp nanostructures (outer diameter D = 22.5 nm, root diameter d1 = 11.3 nm, tip diameter d2 = 1.875nm, the height at the root h1 = 17.5 nm, the height at the tip h2 = 2.5 nm).

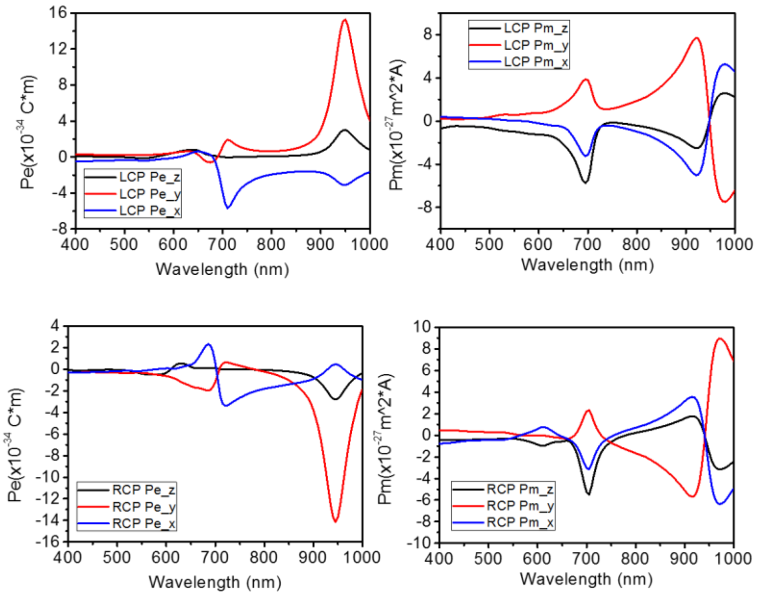

Figure S11. The electric dipole momentum $\mathbf{P_e}$ and magnetic dipole momentum $\mathbf{P_m}$ plotted in their x, y, z components with only real part for the structure in S10.

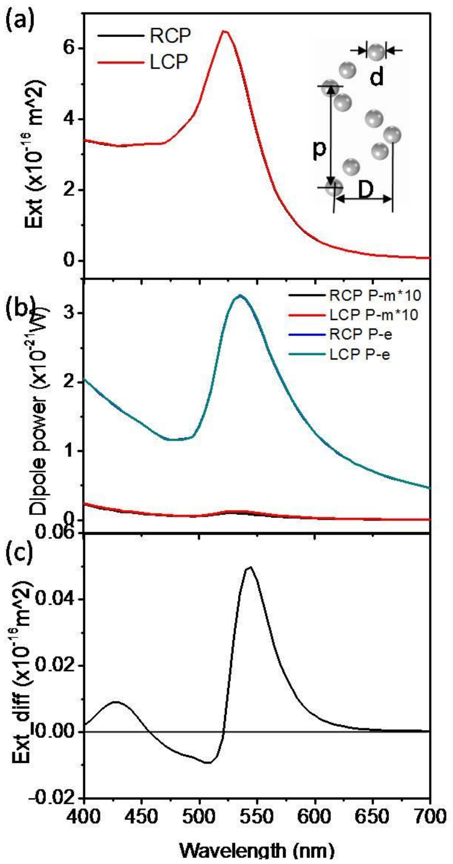

Figure S12. The extinction spectra (a), the dipole power spectra (b) and the extinction different (CD) spectra (c) of the Au nanoparticle helices. (nanosphere diameter: d=10 nm, major diameter:D=34 nm, helical pitch: p= 54nm)[6]

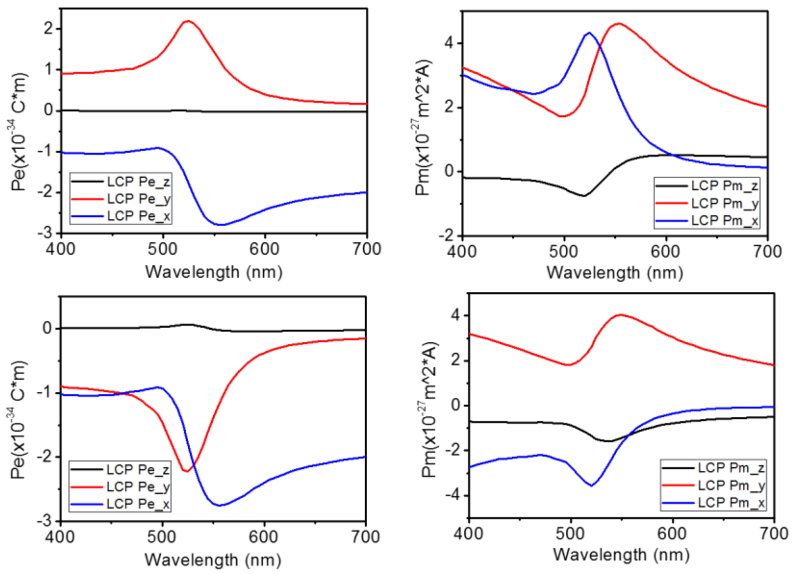

Figure S13. The electric dipole momentum $\mathbf{P_e}$ and magnetic dipole momentum $\mathbf{P_m}$

plotted in their x, y, z components with only real part for the structure in S12.

The scattering spectra of 3D chiral models

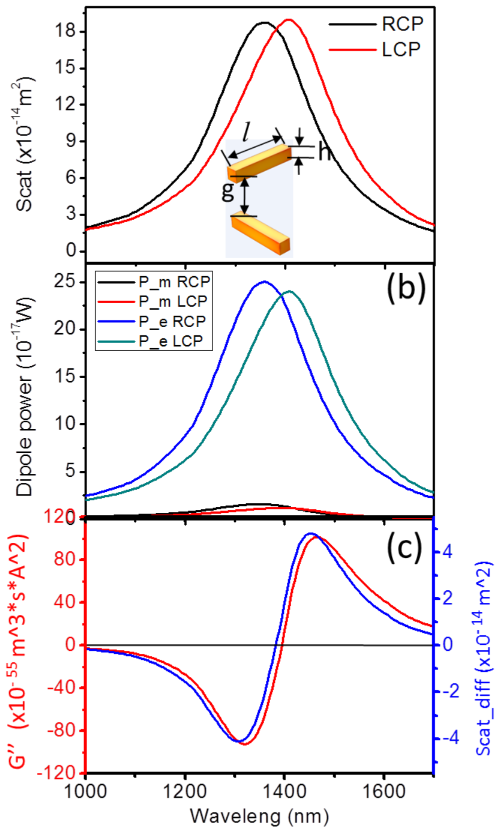

Figure S14. (a) Scattering spectra of the structure (inset) under LCP and RCP excited (length l = 223 nm, height h = 40 nm, width w = 40 nm, gap g = 120 nm). (b) Electric and magnetic dipoles power yielded by the structure under CPL illumination. (c) Imaginary part of the mixed electric and magnetic polarizability (red curve) and scattering difference (CD) of the coupled system (blue curve).

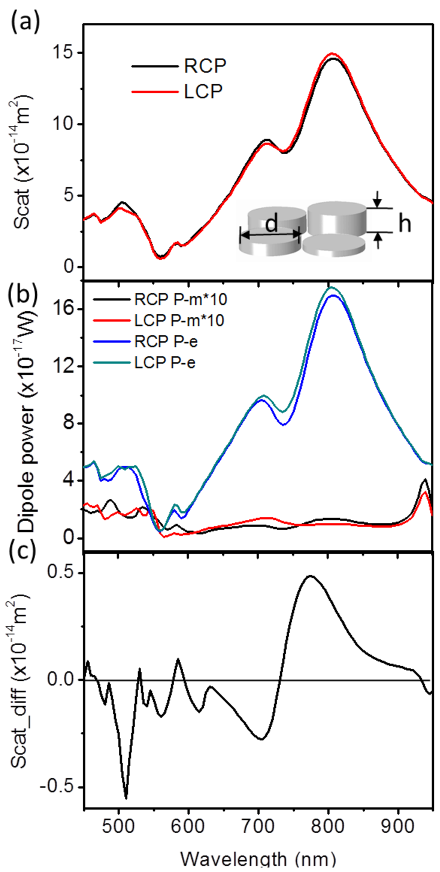

Figure S15. The scattering spectra (a), the dipole power spectra (b)and the scattering different (CD) spectra (c) of the Ag quasi-three-dimensional oligomers.(diameter: d=100nm, h=10 nm, 20 nm, 30 nm, 40 nm, the gap between the oligomers:2 nm).

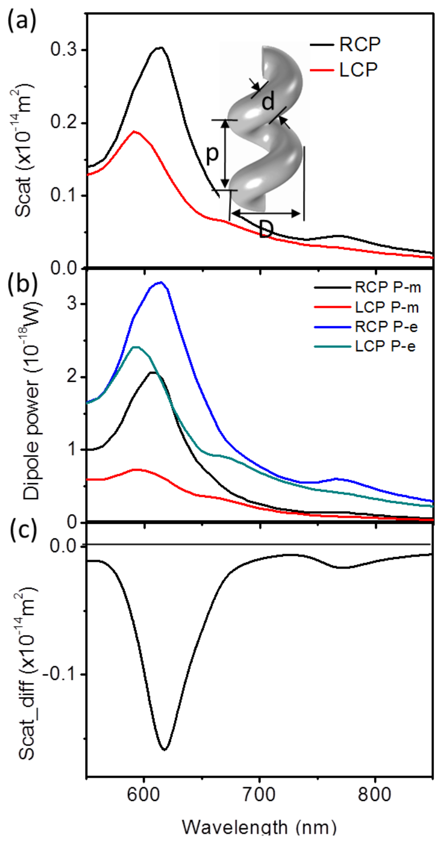

Figure S16. The scattering spectra (a), the dipole power spectra (b) and the scattering different (CD) spectra (c) of the Cu plasmonic nanohelix. (major diameter: D=36 nm, minor diameter: d=28 nm, helical pitch: p= 60 nm).

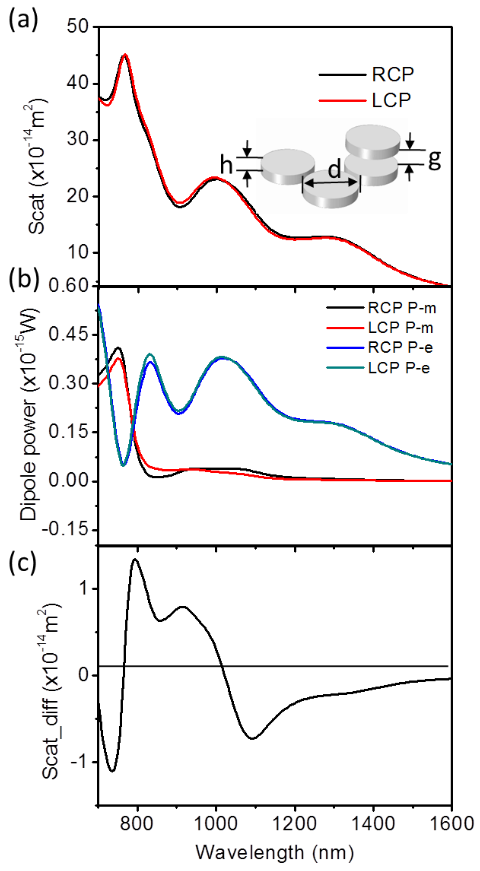

Figure S17. The scattering spectra (a), the dipole power spectra (b) and the scattering different (CD) spectra (c) of the Au 3D chiral plasmonic oligomers. (diameter: d=100 nm, thickness of nanoparticle: h=40nm, the gap between the oligomers in borrom layer: s=20 nm, the gap of two layers: g=70nm).

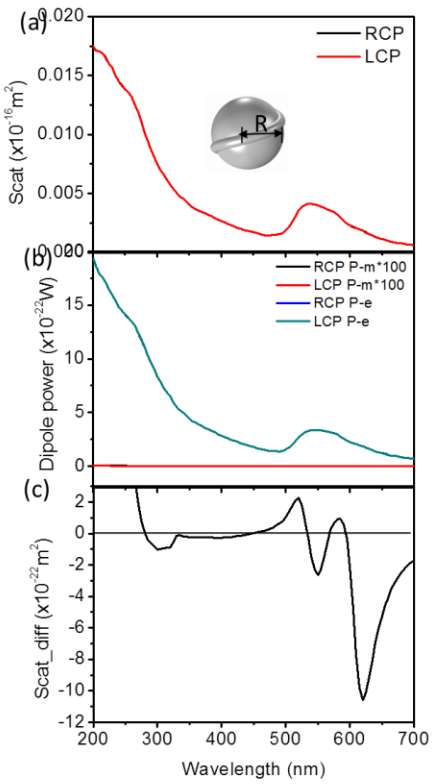

Figure S18. The scattering spectra (a), the dipole power spectra (b) and the scattering different (CD) spectra (c) of the Au chiral nanocrystals.(radius of sphere: R=7 nm, radius of the twiser: 1 nm).

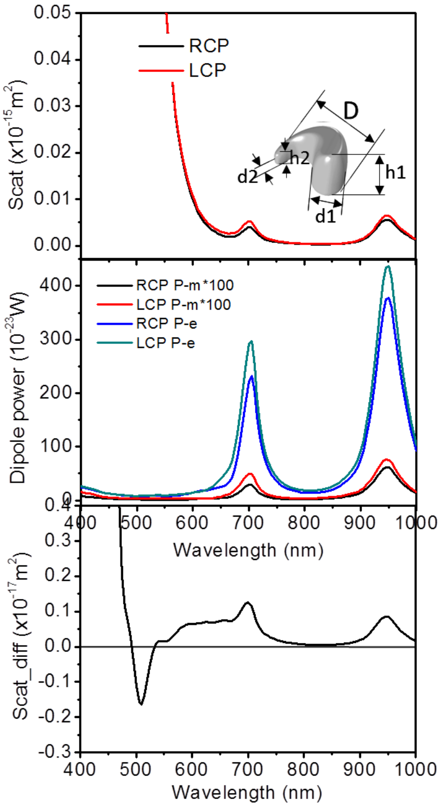

Figure S19. The scattering spectra (a), the dipole power spectra (b) and the scattering different (CD) spectra (c) of the spiral-type ramp nanostructures (outer diameter D = 22.5 nm, root diameter d1 = 11.3 nm, tip diameter d2 = 1.875nm, the height at the root h1 = 17.5 nm, the height at the tip h2 = 2.5 nm).

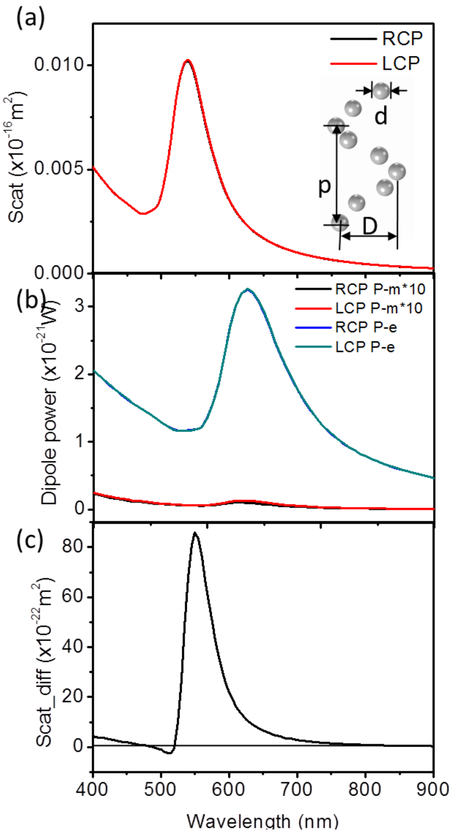

Figure S20. The scattering spectra (a), the dipole power spectra (b) and the scattering different (CD) spectra (c) of the Au nanoparticle helices. (nanosphere diameter: d=10 nm, major diameter:D=34 nm, helical pitch: p= 54nm).

References:

1 Hu, L., Tian, X., Huang, Y., Fang, L. & Fang, Y. Quantitatively analyzing the mechanism of giant circular dichroism in extrinsic plasmonic chiral nanostructures by tracking the interplay of electric and magnetic dipoles. *Nanoscale* **8**, 3720-3728, doi:10.1039/c5nr08527f (2016).

2 Ogier, R., Fang, Y., Svedendahl, M., Johansson, P. & Käll, M. Macroscopic Layers of Chiral Plasmonic Nanoparticle Oligomers from Colloidal Lithography. *ACS Photonics* **1**, 1074-1081, doi:10.1021/ph500293u (2014).

3 Gibbs, J. G., Mark, A. G., Eslami, S. & Fischer, P. Plasmonic nanohelix metamaterials with tailorable giant circular dichroism. *Applied Physics Letters* **103**, 213101, doi:10.1063/1.4829740 (2013).

4 Hentschel, M., Schäferling, M., Weiss, T., Liu, N. & Giessen, H. Three-Dimensional Chiral Plasmonic Oligomers. *Nano Letters* **12**, 2542-2547, doi:10.1021/nl300769x (2012).

5 Fan, Z. & Govorov, A. O. Chiral Nanocrystals: Plasmonic Spectra and Circular Dichroism. *Nano Letters* **12**, 3283-3289, doi:10.1021/nl3013715 (2012).

6 Kuzyk, A. *et al.* DNA-based self-assembly of chiral plasmonic nanostructures with tailored optical response. *Nature* **483**, 311-314, doi:10.1038/nature10889 (2012).
